# Feasibility of individual patient data meta-analyses in orthopaedic surgery

**DOI:** 10.1186/s12916-015-0376-6

**Published:** 2015-06-03

**Authors:** Benoit Villain, Agnès Dechartres, Patrick Boyer, Philippe Ravaud

**Affiliations:** Centre de Recherche Epidémiologie et Statistique, Inserm U1153, Paris, France; Faculté de Médecine, Université Paris Descartes, Sorbonne Paris Cité, Paris, France; Centre d’Epidémiologie Clinique, Hôpital Hôtel-Dieu, Assistance Publique-Hôpitaux de Paris, Paris, France; French Cochrane Centre, Paris, France; Service de Chirurgie Orthopédique, Hôpital Bichat, Assistance Publique-Hôpitaux de Paris, Paris, France; Faculté de Médecine, Université Paris Diderot, Paris, France; Department of Epidemiology, Mailman School of Public Health, Columbia University New York, New York, USA

**Keywords:** Individual patient data, Meta-analysis, Data sharing, Randomized controlled trials, Surgery

## Abstract

**Background:**

The number of individual patient data meta-analyses published is very low especially in surgical domains. Our aim was to assess the feasibility of individual patient data (IPD) meta-analyses in orthopaedic surgery by determining whether trialists agree to send IPD for eligible trials.

**Methods:**

We performed a literature search to identify relevant research questions in orthopaedic surgery. For each question, we developed a protocol synopsis for an IPD meta-analysis and identified all related randomized controlled trials (RCTs) with results published since 2000. Corresponding authors of these RCTs were sent personalized emails that presented a project for an IPD meta-analysis corresponding to one of the research questions, with a link to the protocol synopsis, and asking for IPD from their RCT. We guaranteed patient confidentiality and secure data storage, and offered co-authorship and coverage of costs related to extraction.

**Results:**

We identified 38 research questions and 273 RCTs related to these questions. We could contact 217 of the 273 corresponding authors (79 %; 56 had unavailable or non-functional email addresses) and received 68/273 responses (25 %): 21 authors refused to share IPD, 10 stated that our request was under consideration and 37 agreed to send IPD. Four corresponding authors required authorship and three others asked for financial support to send the IPD. Overall, we could obtain IPD for 5,110 of 33,602 eligible patients (15 %). Among the 38 research questions, only one IPD meta-analysis could be potentially initiated because we could receive IPD for more than 50 % of participants.

**Conclusion:**

The present study illustrates the difficulties in initiating IPD meta-analyses in orthopaedic surgery. Significant efforts must be made to improve data sharing.

**Electronic supplementary material:**

The online version of this article (doi:10.1186/s12916-015-0376-6) contains supplementary material, which is available to authorized users.

## Background

Individual patient data (IPD) meta-analyses (MAs) are generally considered to provide the highest level of evidence [1]. IPD MAs have theoretical advantages over MAs of aggregated data because the use of original source material allows for standardizing analyses across studies and trial results are obtained directly, independent of the quality of reporting [1–3]. Nevertheless, the number of IPD MAs published is very low, with a mean of 49 IPD MAs published each year between 2005 and 2009 [1] as compared with thousands of MAs of aggregated data. The number of IPD MAs is particularly low in surgical domains. In a recent systematic review of IPD MAs, 22 of the 583 IPD MAs published between 2005 and 2012 assessed surgical interventions [4].

The main barrier to performing an IPD MA is the lack of data sharing by researchers. Many researchers are reluctant to share IPD because of patient protection, ownership [5], or the cost [1, 6] to ensure confidentiality and anonymity of patients and archiving the data [1, 7]. Moreover, additional pitfalls, such as the availability of data [8] with practical difficulties related to data sharing many years after the completion of a trial, potentiate the risk of an incomplete data sharing process. There is an evolution in ideas and thinking about data sharing among researchers [9–16], funders [17–19], the Cochrane collaboration [20] and journals [21–25]. However, to our knowledge, no clear data sharing policy has been established by orthopaedic journals or institutions. Thus, data sharing in orthopaedic surgery relies mainly on cooperation from the clinical trialists who generate and maintain these data.

In the present study, we aimed to assess the feasibility of performing IPD MAs in orthopaedic surgery, testing whether trialists agree to share IPD for IPD MAs. We also assessed the conditions that allow for such data sharing.

## Methods

In a first step, we identified orthopaedic clinical research questions that could be evaluated with IPD MAs. Then, using personalized emails, we systematically contacted corresponding authors of randomized controlled trials (RCTs) related to those clinical questions and asked them whether they would agree to share the IPD from their trials for an MA.

### Identification of clinical research questions in orthopaedic surgery

To identify relevant clinical research questions in orthopaedic surgery that could justify an IPD MA, we relied on questions assessed in recently published systematic reviews with MAs of aggregated data.

#### Search of systematic reviews with MAs of aggregated data assessing orthopaedic surgical procedures

On 6 April 2014, we searched MEDLINE via PubMed for systematic reviews published between 1 January 2013 and 31 December 2013, using the search equation reported in Additional file 1. This search equation combined MESH terms related to: orthopaedics and free-text words corresponding to the main orthopaedic surgical procedures; publication type and free-text words corresponding tosystematic reviews and MAs; and a modified version of the Cochrane Highly Sensitive Search Strategy to identify RCTs. In addition, we performed a search of the Cochrane Database of Systematic Reviews (Orthopaedics and Trauma and Rheumatology) to identify Cochrane systematic reviews assessing an orthopaedic surgical procedure and published in 2013. The search results were pooled and duplicate records were removed.

#### Selection of systematic reviews

The title, abstract and full text, when necessary, of all identified references were screened by two authors (BV and PB). We included systematic reviews written in English or French and published in 2013 that assessed an orthopaedic surgical procedure, with no restriction on comparator (usual care, placebo, conservative intervention, pharmacological treatment or other surgical implant or procedure), and including an MA of aggregated data based on two or more RCTs. Systematic reviews and MAs that were withdrawn were excluded, as were those for which the full text was not available.

#### Extraction of clinical research questions from the systematic reviews

Two reviewers (BV and PB) independently extracted all elements of the clinical research question assessed from the full text of the systematic reviews by using the Population, Intervention tested, Comparator and primary Outcomes (PICO) acronym [26]. Then, all clinical questions were classified by anatomical region (e.g., shoulder) and surgical procedure tested (e.g., arthroplasty) to identify any potentially redundant research questions.

### Development of protocol synopsis of IPD MA for each clinical research question

For each clinical research question, we developed a standardized protocol synopsis for an IPD MA, with the background and objectives sections derived from the corresponding systematic review with MA of aggregated data and the methods section based on the *Cochrane Handbook for Systematic Reviews of Interventions* [27]. An example of a protocol synopsis is presented in Additional file 2.

### Identification of RCTs relevant to the clinical research questions

We identified all RCTs included in the systematic reviews with MA of aggregated data for the clinical research questions. All RCTs were selected provided they were written in English or French and results were published since 2000, because for trials with results published before 2000, we anticipated difficulties in contacting corresponding authors and obtaining IPD from these trials. Non-randomized studies and quasi-RCTs were excluded, as were RCTs not indexed in MEDLINE. If corresponding authors were involved in several RCTs corresponding to different clinical research questions, we contacted them for only one clinical question chosen at random. Only the RCTs corresponding to this clinical question were selected.

#### Extraction of characteristics of the selected RCTs

Using a pre-tested standardized form, we extracted the following characteristics from the full text and online Additional files, as well as journal websites:

- Corresponding author: name and email address of the corresponding author. When the email address was not available in the article or not functional, the name was entered in PubMed to search for another study published by the author for which the email address was reported. We also screened the website of the author’s institution to search for an email address.
- Characteristics of the trial: publication date, whether the trial was a single-centre or multicentre trial, location of the study (Africa, Asia, Australia and New Zealand, Europe, North America, South America) and funding source (public, private, both public and private, not reported). The geographic location of the corresponding author’s affiliated institution was used to define the location of the study when the study location was not reported or when the RCT was an international multicentre study. We also extracted the number of patients randomized in each RCT.
- Characteristics of the journal in which the RCT was published: name of the journal, whether it was specialized or general and its impact factor. We classified journals according to whether they were in the 10 highest impact factors for a medical condition according to the Journal Citation Reports. We also recorded whether the journal had a data sharing policy and, if so, whether data sharing was a mandatory condition to publish.

### Contacting the corresponding authors of identified RCTs

Corresponding authors of each RCT were contacted by personalized email to participate in a specific IPD MA project and to provide IPD from their RCT. The email stated that the French Cochrane Centre aimed to initiate collaboration among trialists to perform IPD MAs on important orthopaedic topics and that the first project had as an objective the clinical research question for which the RCT was eligible. We guaranteed protection of patient data and secure storage of datasets. We also systematically asked whether the corresponding author or another colleague wanted to be a co-author of the published IPD MA and whether we should cover costs related to data extraction. The emails were personalized for each trialist and clinical research question, with inclusion of the name of the corresponding author, title, year of publication and journal in which the author’s trial was published, as well as the objective of the IPD MA and a link to the protocol synopsis of the IPD MA corresponding to the research question assessed. The subject of the email was ‘Your article ‘Trial title’, published in ‘Journal’ on ‘Year of publication”. Emails were sent using a dedicated Cochrane address and were signed by an academic orthopaedic surgeon on behalf of the French Cochrane Centre, the French Equator Centre and the Inserm Research Centre U1153. An example of the email sent is available in Additional file 3. Two similar reminders were sent to the authors 15 and 30 days after the first email if we did not receive a response to the initial request.

It has to be noted that our hypothesis was that the rate of positive answers would be low but we expected to be able to perform several IPD meta-analyses as part of a PhD program for the clinical questions for which we would have received a sufficient number of positive answers.

### Statistical analyses

Characteristics of systematic reviews and RCTs were described along with the number and percentage for categorical variables. We compared characteristics of trials and journals for trials for which we had a positive response and a negative or no response to our request for data sharing by two-sided chi-square or Fisher’s exact test, as appropriate, with a type I error level of 0.05. We excluded from this comparison authors who responded that our request was under consideration. Data were analyzed by using R version 2.13.1 [28].

## Results

### Identification of clinical research questions in orthopaedic surgery

Additional file 4 describes the flow of the selection of systematic reviews. Briefly, we screened 418 records and selected 63 systematic reviews with MA of aggregated data corresponding to 38 different clinical research questions described in Table 1. The main characteristics of the 63 systematic reviews are presented in Additional file 5.

**Table 1:** Description of the 38 identified clinical research questions in orthopaedics

Anatomic area	Pathology	Intervention	Comparator	Primary outcomes
Shoulder	Rotator cuff tears	Arthroscopic double-row repair	Arthroscopic single-row repair	Function
	Midshaft clavicular fractures	Surgical treatment	Non-operative treatment	Function
	Proximal humeral fractures in older patients	Surgical treatment	Non-operative treatment	Function
	Osteoarthritis	Total shoulder arthroplasty	Shoulder hemiarthroplasty	Function
Arm	Humeral shaft fracture	Intramedullary nail	Internal fixation with plate	Function
Elbow	Supracondylar fractures in children	Lateral pin fixation	Crossed pin fixation	Function, iatrogenic ulnar nerve injury
Wrist	Distal radial fractures	Anterior ORIF	External fixation	Function, radiographic consolidation
	Distal radial fractures	Anterior ORIF	Posterior ORIF	Function, radiographic consolidation
Hip	Osteoarthritis	Minimally invasive approach	Standard approach	Function, revision rate
	Osteoarthritis	No wound drainage after THA	Wound drainage after THA	Function, wound infection, wound haematoma
	Osteoarthritis	Navigated THA	Conventional arthroplasty	Function, revision rate, dislocation
	Intracapsular fractures	Uncemented hemiarthroplasty	Cemented hemiarthroplasty	Mortality, function, pain at 1 year, revision rate
	Osteoarthritis	Uncemented THA	Cemented THA	Function, revision rate
Hip and knee	Osteoarthritis	Antibiotic impregnated cement	Non-antibiotic-impregnated cement	Postoperative infection rate
Knee	Osteoarthritis	TKA, minimally invasive approach	Standard approach for TKA	Function
	Osteoarthritis	Mobile-bearing TKA	Fixed-bearing TKA	Function, reoperation rate
	Osteoarthritis	TKA without tourniquet	TKA under tourniquet	Function, total blood loss
	Osteoarthritis	Drainage clamping after TKA	TKA conventional drainage	Hb loss, transfusion, function
	Osteoarthritis	No patellar resurfacing in TKA	Patellar resurfacing in TKA	Function, reoperation rate, anterior knee pain
	Osteoarthritis	PCL-retaining TKA	Posterior-stabilized TKA	Function, reoperation rate
	Osteoarthritis	TKA electrocautery of patella	No electrocautery of patella	Function, reoperation rate, anterior knee pain
	Osteoarthritis	Gender-specific TKA	Unisex TKA	Function, reoperation rate, pain,
	Osteoarthritis	Unicompartmental knee arthroplasty	Tibial osteotomy	Function, reoperation rate
	ACL tears	Double-bundle ACL reconstruction	Single-bundle ACL reconstruction	Function, reoperation rate
	ACL tears	Early ACL reconstruction	Delayed ACL reconstruction	Function, reoperation rate
	ACL tears	Allograft ACL reconstruction	Autograft ACL reconstruction	Function, reoperation rate
	Arthroscopic procedures	Arthroscopic procedures without tourniquet	Arthroscopic procedures using tourniquet	Function
Leg	Distal tibial shaft fractures	Intramedullary nailing	Internal fixation with plate	Function, nonunion and malunion rate
	Tibial shaft fractures	Reamed intramedullary nailing	Unreamed intramedullary nailing	Function, nonunion and malunion rate
Ankle	Acute Achilles tendon rupture	Early weight bearing	Delayed weight bearing	Function, rerupture rate
	Ankle fractures	Surgical treatment, biodegradable implants	Surgical treatment, conventional implants	Function, nonunion and malunion rate
Foot	Calcaneal fractures	Surgical treatment	Non-operative treatment	Function, chronic pain
Spine	Cervical disc disease	Cervical disc arthroplasty	Cervical interbody fusion	Function, pain
	Osteoporotic vertebral fractures	Percutaneous vertebroplasty	Conservative treatment	Function, pain, quality of life
	Osteoporotic vertebral fractures	Bilateral pedicular kyphoplasty	Unilateral pedicular kyphoplasty	Function, pain, quality of life
	Lumbar disc disease	Lumbar disc arthroplasty	Lumbar interbody fusion	Function, pain
	Thoracolumbar burst fractures	Fracture fixation associated to fusion	Fracture fixation alone	Function, pain, quality of life
	Thoracolumbar burst fractures	Surgical treatment	Conservative treatment	Function, pain, quality of life

ACL: Anterior cruciate ligament; Hb: Haemoglobin; ORIF: Open reduction and internal fixation; PCL: Posterior cruciate ligament; THA: Total hip arthroplasty; TKA: Total knee arthroplasty

### Identification of RCTs corresponding to the clinical research questions

**Fig. 1:**
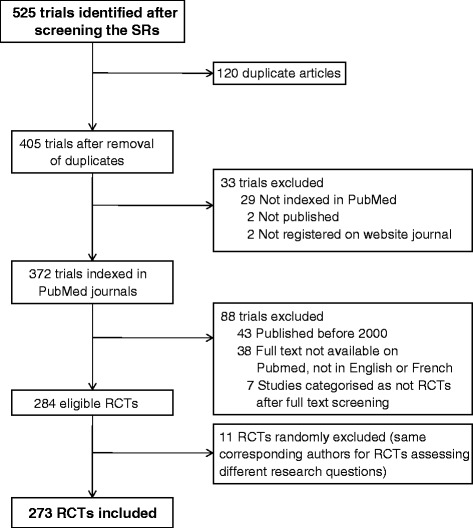
Flow chart of the selection of randomized controlled trials (RCTs) corresponding to the 38 clinical research questions

From the full text of these 63 systematic reviews, we identified 525 trials and selected 284 eligible trials 11 were further excluded because the corresponding authors were involved in several RCTs assessing different research questions, so our study was based on 273 RCTs (Fig. 1).

#### Characteristics of the selected RCTs

The main characteristics of the RCTs are summarized in Table 2. Briefly, 183 RCTs (67 %) were single-centre trials; 60 (22 %) were multicentre trials, with unknown status for 30 trials (11 %). Most of the trials took place in Europe, North America and Asia (39 %, 29 % and 29 %, respectively). The funding source was reported for 53 % of RCTs and was public for 35 %. Forty-two percent of RCTs were published between 2010 and 2013. Most of the RCTs (95 %) were published in specialized journals. Only 32 (12 %) were published in journals that had a clearly described data sharing policy.

**Table 2:** General characteristics of the randomized controlled trials (RCTs) related to the clinical research questions (n = 273)

Characteristics	Number of RCTs (%)
	(n = 273)
Trial characteristics	
*Study design*	
Single-centre	183 (67 %)
Multicentre	60 (22 %)
Not reported	30 (11 %)
*Location*	
Europe	106 (39 %)
North America	80 (29 %)
Asia	78 (29 %)
Australia and New Zealand	9 (3 %)
*Funding*	
Public	96 (35 %)
Private	49 (18 %)
Not reported	128 (47 %)
*Year of publication*	
2000–2004	46 (17 %)
2005–2009	112 (41 %)
2010–2013	115 (42 %)
Journal of publication characteristics	
*Type of journal*	
Specialized	260 (95 %)
Generalist	13 (5 %)
*Top 10 impact factor of each specialty*	
No	156 (57 %)
Yes	117 (43 %)
*Journal data sharing policies*	
No policy	241 (88 %)
Incentive measures	24 (9 %)
Mandatory	8 (3 %)

### Data sharing request

**Fig. 2:**
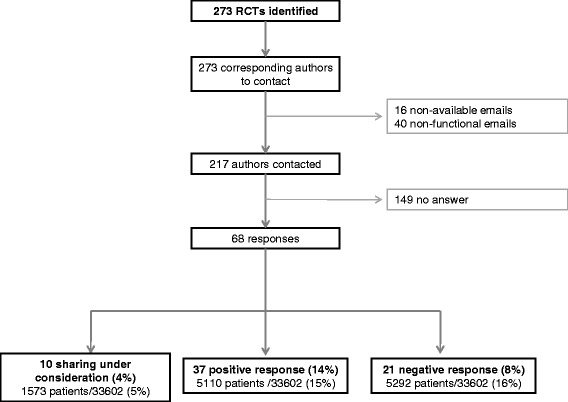
Flow chart of data sharing request process

The email address of the corresponding author was available and functional for 217 of the 273 RCTs (79 %). We received 68 responses (Fig. 2). In total, 37 authors, corresponding to 14 % of the RCTs, agreed to send IPD: 30 without any conditions, four requiring co-authorship and three asking for financial support to send the IPD. Ten additional authors said that our request was under evaluation by the research team or sponsor and 21 authors refused to participate and to send IPD. Authors who refused to send IPD did so because of no access to the data (n = 8), ethical concerns (n = 5), lack of time (n = 5) and no reason (n = 3).

#### Feasibility of IPD MAs in orthopaedic surgery

**Fig. 3:**
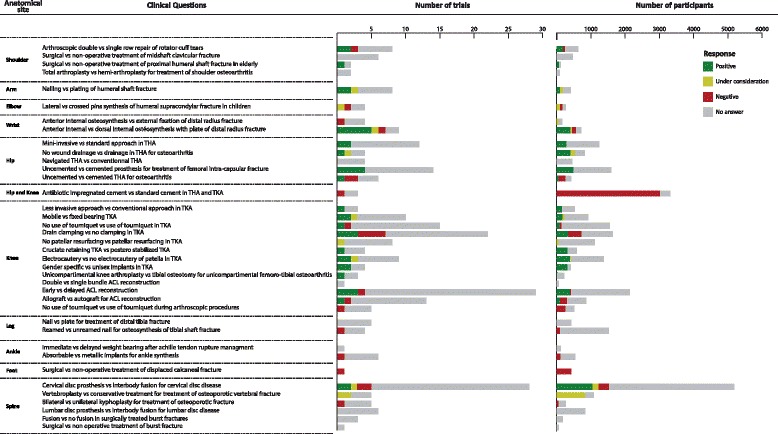
Distribution of answers to request for data sharing by clinical questions. ACL: Anterior cruciate ligament; THA: Total hip arthroplasty; TKA: Total knee arthroplasty

Overall, we obtained agreement to receive IPD for 5,110 of 33,602 eligible patients (15 %). We obtained agreement to receive IPD for more than 50 % of participants for only one of the 38 clinical research questions. This question concerned comparing external fixation and internal anterior fixation for distal radial fracture with 399 participants (five studies) among 712 eligible (nine studies) (56 %) (Fig. 3).

#### Comparison of characteristics of RCTs with a positive response and negative or no response to the request for data sharing

We did not identify any factor significantly associated with a positive response to our request for data sharing (Table 3). We received a positive response for 19/176 (11 %) single-centre trials, 11/57 (19 %) multicentre trials (*P =* 0.07), 20/117 (17 %) trials published in the top 10 journals (with the highest impact factor) and 17/146 (12 %) trials not published in the top 10 journals (*P =* 0.21). We received a positive response for 2/30 (7 %) trials published in journals with data sharing policies and 35/233 (15 %) trials published in journals without data sharing policies (*P =* 0.27).

**Table 3:** Characteristics of RCTs with a positive response and negative or no response to the request for data sharing

Characteristics	Data sharing request^a^ (n = 263)		*P* value
	n (%)		
	Positive response	Negative or no response	
	37 (14%)	226 (86%)	
RCT characteristics			
*Study design*			0.07
Single-centre (n = 176)	19 (11%)	157 (89%)	
Multicentre (n = 57)	11 (19%)	46 (81%)	
Not reported (n = 30)	7 (23%)	23 (77%)	
*Location*			0.11
Europe (n = 105)	20 (19%)	85 (81%)	
North America (n = 71)	9 (13%)	62 (87%)	
Asia (n = 78)	6 (8%)	72 (92%)	
Australia and New Zealand (n = 9)	2 (22%)	7 (78%)	
*Funding*			0.19
Public (n = 91)	8 (9%)	83 (91%)	
Private^b^ (n = 45)	7 (16%)	38 (84%)	
No response (n = 127)	22 (17%)	105 (83%)	
*Year of publication*			0.57
2000–2004 (n = 44)	4 (9%)	40 (91%)	
2005–2009 (n = 109)	16 (15%)	93 (85%)	
2010–2013 (n = 110)	17 (15%)	93 (85%)	
Journal of publication characteristics			
*Journal of publication*			0.68
Specialized (n = 251)	35 (14%)	216 (86%)	
Generalist (n = 12)	2 (17%)	10 (83%)	
*Journal data sharing policies*			0.27
Yes (support or mandatory) (n = 30)	2 (7%)	28 (93%)	
No (n = 233)	35 (15%)	198 (85%)	
*Top 10 impact factor in each specialty*			0.21
Yes (n = 117)	20 (17%)	97 (83%)	
No (n = 146)	17 (12%)	129 (88%)	

^a^Ten trials excluded because our request was being evaluated by the research team or sponsor at the time of statistical analysis; ^b^total number (n = ) used for calculating the percentage

## Discussion

In this study, we requested IPD from corresponding authors for 273 RCTs in order to perform 38 IPD MAs covering different orthopaedic clinical research questions. Our results highlight the difficulty in performing IPD MAs. We could contact only 79 % of the identified corresponding authors despite additional searches on PubMed and the website for the author’s affiliation. The response rate was only 25 % (68/273 authors), with only 14 % (37/273) agreeing to participate in the IPD MA project. Because of this low rate of participation, only one IPD MA among the 38 planned could be potentially initiated.

### Strengths and weaknesses

To the best of our knowledge, this study was the first to assess the feasibility of performing a large number of IPD MAs in real conditions. We had a pragmatic approach in adopting the point of view of researchers willing to initiate IPD MAs. Emails were personalized to each trialist and to each research question, with the protocol synopsis corresponding to the specific clinical question provided. We focused on orthopaedics because the number of clinical trials performed in this field is low [29–31] as compared with other specialties, so cooperation of trialists and data sharing are crucial to provide high-level evidence regarding the efficacy of interventions.

Some potential limitations should be discussed. We attempted to contact corresponding authors by email only. The rate of response could have been higher if we had contacted authors by telephone or postal mail. Also, it could have been higher with the collaboration of learned societies. We were able to contact only 79 % of investigators because of invalid email addresses, which can be explained by their moving to another institution. Author identification initiatives and online research profiles such as ResearchGate could help identify contact information of investigators [32]. The positive responses we received were an agreement to share IPD, but with no guarantee to finally obtain the IPD, so we may have overestimated the real number of datasets available for our IPD MA projects. Finally, Hannink et al. [4] identified an IPD MA published in 2011 assessing a question close to one of our 38 questions, which may explain the lack of positive response for this question [33].

### Comparison with other studies

Some authors previously surveyed trialists’ opinions on data sharing, with most respondents in favour of sharing IPD [34, 35]. The difference from our results may be due to the fact that surveyed trialists were corresponding authors of trials recently published in general journals with the highest impact factor [34], some of these journals having adopted strong data sharing policies [22]. Also, our pragmatic approach may explain the difference. There is probably a gap between favouring data sharing and actually sending the IPD. There are some rare examples concerning a particular case reporting the difficulties encountered in obtaining IPD [36–39] including for performing IPD MAs [38, 39]. Jaspers et al. could not initiate an IPD MA on proton magnetic resonance spectroscopy in neonatal hypoxic-ischemic encephalopathy, receiving five favourable responses among 18 contacts [38]. The Cochrane Epilepsy Group recently reported their difficulties in performing review updates, with IPD obtained for only 299 participants (four studies) among the 7,811 eligible (37 studies identified) [39].

### Possible explanations and implications

The arguments given by corresponding authors for refusing to send IPD agree with the barriers to data sharing described in the literature: concerns about protecting patient confidentiality and anonymity [1, 7, 40], lack of time and costs [1], and unusable datasets [1]. Practical solutions and guidelines to circumvent those obstacles have been proposed [12, 14, 40, 41], but significant efforts must be made to raise the awareness of the surgical community about the benefits of data sharing. Data sharing is a necessary condition to perform IPD meta-analyses, recognized as providing the highest level of evidence. This type of study is of great interest in domains for which randomized controlled trials are difficult to perform, such as in surgery to increase precision and power, to improve external validity and to perform subgroup analyses. Nevertheless, to be relevant, an IPD meta-analysis should be based on all relevant evidence and not on a subset (potentially biased) of eligible trials [1, 2, 8]. In this study, we focused on orthopaedics but it is likely that other surgical domains may be concerned. In a recent methodological review of 583 identified IPD meta-analyses, only 22 (4 %) concerned a surgical intervention [4].

Things are moving with a push toward more data sharing among researchers and some journals [21, 22] that require authors to make the relevant anonymized patient-level data available on reasonable request and to provide a data sharing statement in each article. Major academic funders as well as some pharmaceutical companies are also adopting policies to support this sharing of data. This movement should also spread to surgical domains. Specialized journals, learned societies, funders as well as research leaders have a major role to play to help improve data sharing in surgical domains. Nevertheless, there are practical issues as sharing data 10 years after the completion of a trial may be difficult related to availability and format of data, and because there is no patient consent. The responsibility for cleaning, storing and sharing databases is so far supported by individual researchers. As outlined in an article published in *Current Biology*, in the long term, research data cannot be reliably preserved by individual researchers [32]. Data cleaning, storage and export in a suitable format represent an important burden for researchers with no specific funding dedicated to this process. Also, investigators may move to another institution, which presents difficulties in contacting them. Therefore, institutions and funders should have the responsibility for data storage. A major change could be obtained by the establishment of central repositories, domain by domain, with close collaboration between researchers, funders and learned societies to securely deposit IPD in a standardized format promptly after trial completion. The development of such repositories is ongoing in some domains, such as rheumatology with the Osteoarthritis Trial Bank [42]. Patient consent to deposit anonymous data on a repository and standardization of data could be planned along with the clinical trial to facilitate the process. Finally, as recently outlined by Ioannidis, the current system does not reward enough data sharing [43]. Additional value should be given to researchers who agree to share their data.

## Conclusions

The present study illustrates the difficulties in initiating IPD MAs in orthopaedic surgery. Even under the most favourable conditions (recent trials, request by the French Cochrane Centre, co-authorship, coverage of costs related to data extraction), the number of trials for which IPD could be obtained was low. Significant efforts must be made by the different players in medical research to improve data sharing in the surgical community.

## Key messages

This study outlines the practical difficulties in performing IPD MAs in orthopaedics.

- We obtained a response rate of 25 % to our request for IPD, with only 14 % of authors agreeing to send IPD.
- Overall, we obtained agreement for IPD sharing concerning 5,110 of 33,602 eligible patients.
- We obtained agreement to receive IPD for more than half of participants for only one of the 38 IPD MA projects that could have been initiated.

## Additional files

Additional file 1:
**Search equation of systematic reviews with meta-analysis of aggregated data assessing orthopaedic surgical procedures.**
Additional file 2:
**Example of protocol synopsis for individual patient data meta-analysis.**
Additional file 3:
**Description of emails sent to the corresponding author to ask for individual patient data.**
Additional file 4:
**Flow chart describing the selection process of systematic reviews with meta-analysis of aggregated data assessing orthopaedic surgical procedures.**
Additional file 5:
**Main characteristics of the systematic reviews with meta-analysis of aggregated data published in 2013 (n = 63).**

## Abbreviations

IPD: Individual patient data
MA: Meta-analysis
PICO: Population, Intervention tested, Comparator and primary Outcomes
RCT: Randomized controlled trial
