# Supplementary material for: Feasibility of individual patient data meta-analyses in orthopaedic surgery
Source: BMC Med. 2015 Jun 3;13:131. doi: 10.1186/s12916-015-0376-6 (PMC4464630; doi:10.1186/s12916-015-0376-6)
Supplement: Additional file 1: — Search equation of systematic reviews with meta-analysis of aggregated data assessing orthopaedic surgical procedures. [file 12916_2015_376_MOESM1_ESM.doc]

# Additional file 1: Search equation of systematic reviews with meta-analysis of aggregated data assessing orthopaedic surgical procedures

| "Search (((((((((((((((((((((((((((((((fractures, bone[MeSH Terms]) OR ""fracture fixation""[MeSH Terms]) OR fracture[Title/Abstract]) OR fractures[Title/Abstract]) OR bone nails[MeSH Terms]) OR bone plates[MeSH Terms]) OR bone screws[MeSH Terms]) OR bone wires[MeSH Terms]) OR external fixators[MeSH Terms]) OR osteoarthritis[MeSH Terms]) OR arthroplasty[MeSH Terms]) OR arthroplasty[Title/Abstract]) OR ""hemiarthroplasty""[MeSH Terms]) OR hemiarthroplasty[Title/Abstract]) OR joint prosthesis[Title/Abstract]) OR hip replacement[Title/Abstract]) OR knee replacement[Title/Abstract]) OR ankle replacement[Title/Abstract]) OR shoulder replacement[Title/Abstract]) OR elbow replacement[Title/Abstract]) OR finger replacement[Title/Abstract]) OR arthroscopy[MeSH Terms]) OR arthroscopy[Title/Abstract]) OR rotator cuff[MeSH Terms]) OR rotator cuff[Title/Abstract]) OR arthroscopic[Title/Abstract]) OR tendons[MeSH Terms]) OR tendon[Title/Abstract]) OR ligament reconstruction[Title/Abstract]) OR ligament injury[Title/Abstract]) OR ligament injuries[Title/Abstract]) OR joint instability[MeSH Terms] Sort by: PublicationDate"  Combined with: |
| --- |

((((((("review"[Publication Type] OR "overview"[tiab]) OR meta analysis[pt]) OR "meta-analyses"[tiab]) OR "meta-analysis"[tiab]) OR "systematic reviews"[tiab]) OR "systematic review"[tiab]) AND ("2013/01/01"[PDAT] : "2013/12/31"[PDAT])) AND (((((("randomized controlled trial"[Publication Type] OR "controlled clinical trial"[Publication Type]) OR randomized[Title/Abstract]) OR randomly[Title/Abstract]) OR trial[Title/Abstract]) OR groups[Title/Abstract]) NOT ("animals"[MeSH Terms] NOT "humans"[MeSH Terms]))
