# Supplementary material for: Feasibility of individual patient data meta-analyses in orthopaedic surgery
Source: BMC Med. 2015 Jun 3;13:131. doi: 10.1186/s12916-015-0376-6 (PMC4464630; doi:10.1186/s12916-015-0376-6)
Supplement: Additional file 2: — Example of protocol synopsis for individual patient data meta-analysis. [file 12916_2015_376_MOESM2_ESM.doc]

# Additional file 2: Example of protocol synopsis for individual patient data meta-analysis

**Comparison of internal and external fixation of distal radius fractures:**

**An individual patient data meta-analysis**

**Background:**

Fractures of the distal radius comprise the largest portion of hand and forearm fractures, accounting for more than one-sixth of all fractures treated in emergency departments.

The common fixation techniques for unstable distal radius fractures that cannot be treated with casting include closed reduction and external fixation with or without Kirschner wires and open reduction and internal fixation (ORIF) with dorsal and/or volar plates. Bridging external fixation relies on ligament taxis to obtain and maintain fracture alignment. Excellent results have been described for both techniques and no conclusive evidence has been published favouring ORIF with a volar locking plate over bridging external fixation or vice versa.

**Objectives:**

Therefore, the aim of the current study is to compare the efficacy and safety of bridging external fixation with volar plating in patients with unstable distal radius fractures.

**Methods:**

This protocol of IPD meta-analysis project will be registered on PROSPERO.

A steering committee and a collaborative group will formalize this IPD meta-analysis project. The steering committee will supervise the research team and will approve and agree on all decisions made and methods applied, provide the content of the license agreement between data deliverers and the research team, and ensure handling of data and provide safe data storage.

*Criteria for considering studies for this review*

*Types of studies:*

We will include published and unpublished randomised controlled trials that compared internal and external fixation of unstable distal radius fractures. Every effort will be made to obtain individual participant data (IPD) for trials meeting the selection criteria and eligible trial will be excluded from the analysis if IPD are not available.

*Types of participants:*

We will include male and female participants over the age of 18 who had been diagnosed unstable distal radius fractures.

*Types of interventions:*

Intervention group: open reduction and internal fixation with volar plates

Control group: closed reduction and external fixation with or without Kirschner wires

*Types of outcome measures*

*Primary outcomes:*

Functional outcome (e.g. the Disabilities of the Arm, Shoulder, and Hand (DASH) score, or other functional score)

Radiographic consolidation

*Secondary outcomes:*

Reoperation rate

Grip strength

Complications: minor complications included transient extensor tendon irritation, superficial infections, and finger stiffness.

Major complications included loss of reduction, mal union, and nonunion as well as deep infection, neuropathy, and tendon rupture.

We will extract outcome data at the following time periods: short-term follow-up (up to six weeks following treatment); intermediate follow-up (more than six weeks and up to six months after the end of treatment) and long-term (longer than six months after the end of treatment)

*Search methods for identification of studies*

We will search the Cochrane Central Register of Controlled Trials, MEDLINE and EMBASE with no language restrictions. In addition, we will search clinicaltrials.gov and the WHO International Clinical Trials Registry Platform for registered RCTs. Finally, we will perform a manual search in the grey literature, searching unpublished studies in annual meeting of the European Federation of National Associations of Orthopaedics and Traumatology (EFORT), the American Association of Orthopedic Surgeon (AAOS) and reference lists of articles and reviews for possible relevant studies.

*Data collection and analysis*

*Selection of studies:*

Title, abstract and full text when necessary of any RCT identified by the search strategy will be scanned independently by two authors (BV and PB).The inclusion decisions will be made independently by the two authors. A third author (AD) will be contacted for consensus when discrepancy between the two authors could not be resolved by discussion.

*Data sharing and publication policies:*

Trialists of included studies will be invited to collaborate in accordance with section 18.2 of the *Cochrane Handbook*. One trialist of each included study will have the opportunity to participate in the meta-analysis and will be offered authorship.

*Data extraction and management:*

Trialists will be asked to provide the trial protocol and then IPD for all randomised participants to be used in the review. We will adopt data security measures to ensure data protection. To ensure that the data cannot be violated or tracked, all data sets will be stored securely and anonymously at the *Centre de recherché épidémiologie et statistiques* Sorbonne Paris Cité, INSERM U1153. After discussion with the collaborative group, the steering committee will state on which data will be analyzed (minimal dataset).

*Planned comparisons:*

1/ Internal versus external fixation of distal radius fractures

2/ If possible, studies will be categorized into different subgroup analyses according to fracture type and implants characteristics

*Data checking and cleaning:*

Standard checks will be applied to all incoming trials, including checks for missing values, data validity and consistency across variables. In addition, data received from the trialists will be checked for consistency with the trial protocol and with results presented in the paper.

*Assessment of risk of bias in included studies:*

The methodological quality of all included RCTs will be independently assessed by two authors (BV and PB) using The Cochrane Collaboration’s ‘Risk of bias’ tool. Any difference in evaluation between the two authors will be resolved by discussion and asking AD for consensus when necessary.

*Measures of treatment effect*

Binary outcomes: We will calculate the relative risk (RR) and its 95% confidence interval (CI).

Continuous outcomes: When the same scale has been used in all included studies, we will calculate the standardized mean differences (SMD) and their 95% CI. Where different scales are used for the same outcome, effect sizes will be calculated separately for each scale.

Change versus endpoint data: we will use change from baseline data.

*Dealing with missing data*

The main analyses of all endpoints, subsets and subgroups will be carried out on the basis of the intention-to-treat principle; that is, participants will be analyzed according to their allocated treatment, irrespective of whether they received that treatment or not, but they will be based on complete cases and no attempt will be made to impute missing data. In our request for the raw data from the included trials it will be made clear that data are needed for all randomised participants.

*Data analysis:*

Data from the included studies will be analyzed using a two-step approach: at the first step, we will analyze the IPD for each trial, separately and at the second step we will combine the estimates of effect across studies in meta-analysis. Analysis plan and study results will be discussed with the collaborative group and validated by the steering committee during collaborative group meetings; one kick of meeting and two other meeting are planned.

*Assessment of heterogeneity:*

Descriptive comparisons between studies will be conducted to assess between-study differences. We will analyze treatment effects using a random-effects model. The I² statistic will be used to estimate the total variation across studies.

If possible, we will conduct subgroup analyses according to potentially significant factors after validation by the steering committee.

*Sensitivity analysis:*

Sensitivity analysis will be performed, separately, by excluding studies assessed as being at high risk of bias on one or more of the ’Risk of bias’ items: sequence generation, allocation concealment or completeness of outcome data.
