# Supplementary material for: Feasibility of individual patient data meta-analyses in orthopaedic surgery
Source: BMC Med. 2015 Jun 3;13:131. doi: 10.1186/s12916-015-0376-6 (PMC4464630; doi:10.1186/s12916-015-0376-6)
Supplement: Additional file 3: — Description of emails sent to the corresponding author to ask for individual patient data. [file 12916_2015_376_MOESM3_ESM.doc]

**Additional file** **3: Description of emails sent to the corresponding author to ask for individual patient data**

**From:** patrick.boyer@cochrane.fr [[mailto:patrick.boyer@cochrane.fr]](mailto:%5Bmailto:patrick.boyer@cochrane.fr%5D)

**Sent:** mardi 17 juin 2014 09:54
**To:**

**Subject:** Your article '*Trial title*', published in ‘*Journal’* on ‘*Year of publication’*

| Dear Dr ‘*Corresponding author name*’, |
| --- |
| We are contacting you because you are the corresponding author of the study titled "*Trial title*", published in ‘*Journal’* on ‘*Year of publication*’. |
| The French Cochrane Center in collaboration with the Center for Epidemiological and Statistical Research (CRESS, INSERM U1153) and the French Equator Center is working on a project to perform meta-analyses of important orthopedic topics using individual patient data (IPD). IPD meta-analyses pool the data shared by the authors of applicable trials and require team collaboration. We aim to initiate collaborative projects between the French Cochrane Center and authors of selected randomized controlled trials (RCTs), such as you. |
| Our first project is to perform a meta-analysis of IPD to assess ‘*Objective clinical question*’. Because your study is eligible for this meta-analysis, we wonder whether you would be willing to participate in this initiative. We invite you to read the protocol synopsis of the meta-analysis attached to this email and let us know if you agree to share the Individual Patient Data from your study. |
| [Click here to upload the protocol synopsis](http://www.epidemio-hoteldieu.com/Envoi_Benoit_Villain/protocol/Use_of_tourniquet_in_total_knee_arthroplasty.pdf) |
| We guarantee the use of data security measures to ensure protection of the patient data, and all datasets will be stored securely. All data received will be analyzed as planned in the protocol. Finally, you or one of your colleagues will have the opportunity to participate in the meta-analysis. We cannot guarantee that results will be published (if we do not receive sufficient data), but we promise co-authorship with publication. |
| Please, let us know:  1. If you accept to share the Individual Patient Data from your RCT with us  2. If you have any conditions related to this data sharing (eg, reimbursement of costs related to extraction of individual patient data) |
| We would very much appreciate it if you could respond to this email before june, 27 2014. |
| Please do not hesitate to contact us if you have any question or concerns. |
| Thank you for your time and consideration. |
| Sincerely yours |
| Patrick Boyer, M.D., Ph.D.  Orthopedic Surgeon  On behalf of the scientific committee composed of Philippe Ravaud M.D., Ph.D., French Cochrane Centre, French Equator Centre, member of the Consort group, Inserm U1153, Paris Descartes University, Hôtel-Dieu hospital (AP-HP), Benoît Villain M.D. French Cochrane Center, Inserm U1153, Agnes Dechartres M.D., Ph.D., French Cochrane Centre, French Equator Centre, Inserm U1153, Paris Descartes University and Patrick Boyer M.D., Ph.D., French Cochrane Center, Bichat hospital. |
| 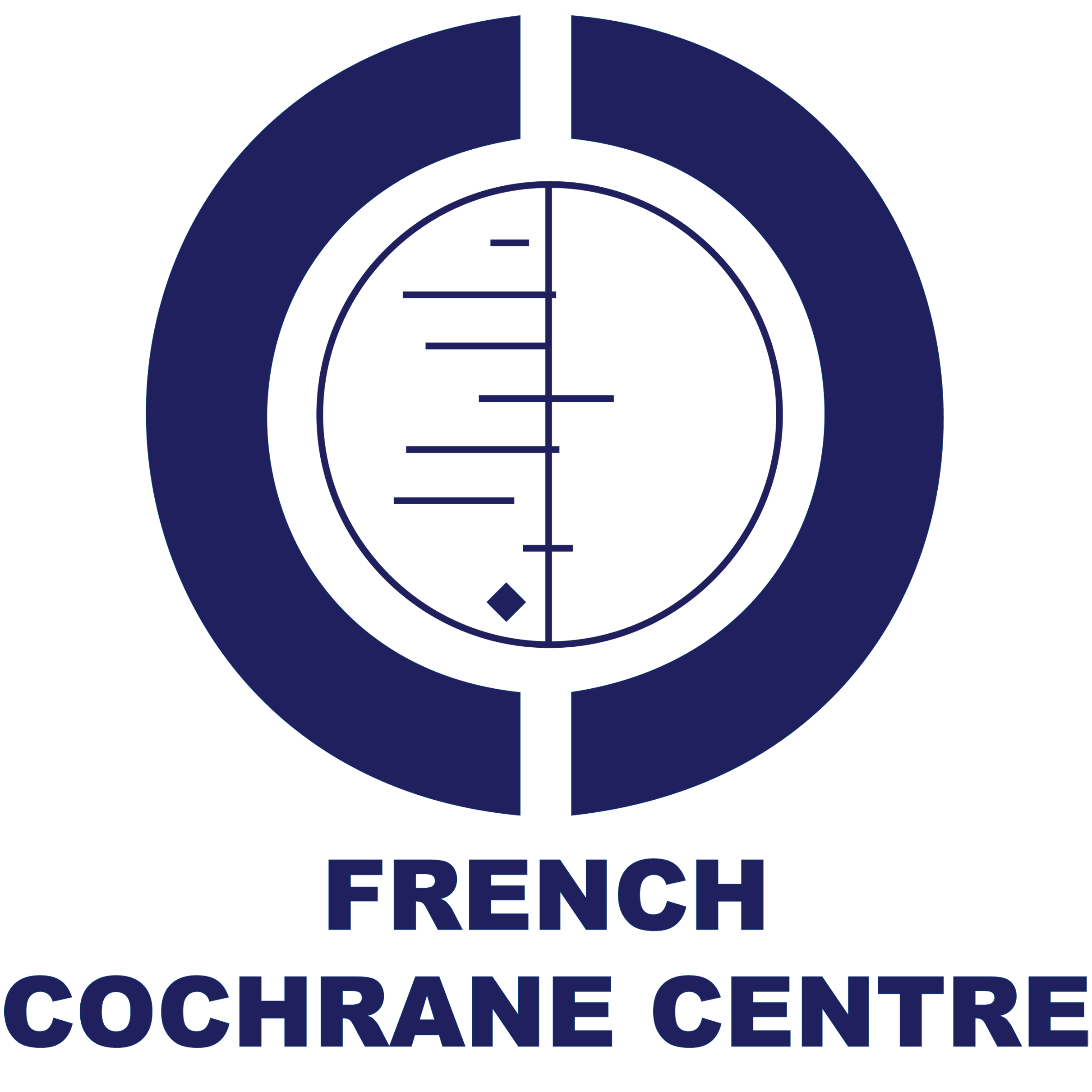    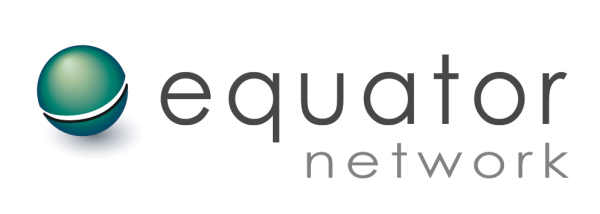     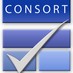 |
| 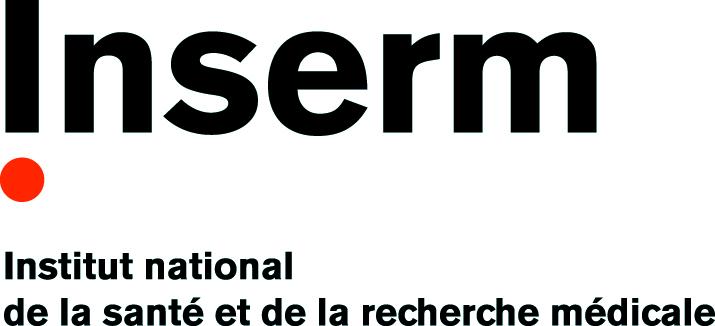 |
