# Supplementary material for: Feasibility of individual patient data meta-analyses in orthopaedic surgery
Source: BMC Med. 2015 Jun 3;13:131. doi: 10.1186/s12916-015-0376-6 (PMC4464630; doi:10.1186/s12916-015-0376-6)
Supplement: Additional file 4: — Flow chart describing the selection process of systematic reviews with meta-analysis of aggregated data assessing orthopaedic surgical procedures. [file 12916_2015_376_MOESM4_ESM.ppt]

## Slide 1
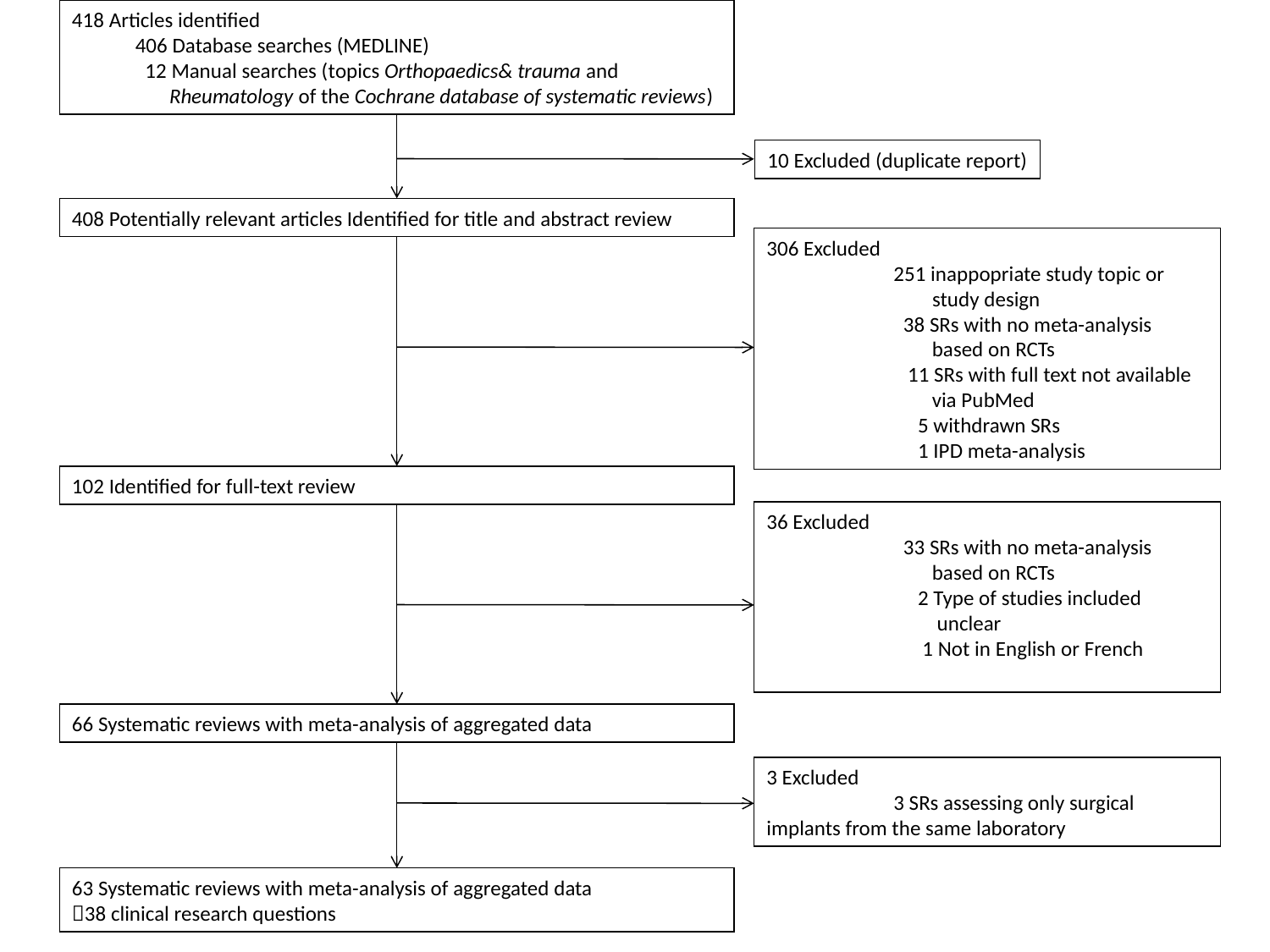

418 Articles identified
 406 Database searches (MEDLINE)
 12 Manual searches (topics Orthopaedics& trauma and
 Rheumatology of the Cochrane database of systematic reviews)
10 Excluded (duplicate report)
408 Potentially relevant articles Identified for title and abstract review
306 Excluded
	251 inappopriate study topic or 	 study design
	 38 SRs with no meta-analysis
 based on RCTs
 11 SRs with full text not available
 via PubMed
	 5 withdrawn SRs
	 1 IPD meta-analysis
102 Identified for full-text review
36 Excluded
	 33 SRs with no meta-analysis
 based on RCTs
	 2 Type of studies included
 unclear
 1 Not in English or French
66 Systematic reviews with meta-analysis of aggregated data
3 Excluded
	3 SRs assessing only surgical 	implants from the same laboratory
63 Systematic reviews with meta-analysis of aggregated data
38 clinical research questions
