# Supplementary material for: Feasibility of individual patient data meta-analyses in orthopaedic surgery
Source: BMC Med. 2015 Jun 3;13:131. doi: 10.1186/s12916-015-0376-6 (PMC4464630; doi:10.1186/s12916-015-0376-6)
Supplement: Additional file 5: — Main characteristics of the systematic reviews with meta-analysis of aggregated data published in 2013 (n = 63). [file 12916_2015_376_MOESM5_ESM.doc]

# Additional file 5: Main characteristics of the systematic reviews with meta-analysis of aggregated data published in 2013 (n=63)

| **Characteristics** | **No. of systematic reviews (%) (n=63)** |
| --- | --- |
| **Systematic review**  *Type of study analysed*  Only RCTs  RCTs and non-randomised studies  RCTs and quasi-RCTs | 45 (71%)  13 (21%)  5 (8%) |
| *Surgical procedures assessed*  Arthroplasty  Knee  Spine  Hip  Shoulder  Hip and knee  Fracture management  Spine (osteoporotic and traumatic)  Arm (humerus)  Shoulder (proximal humerus and clavicle)  Wrist (distal radial fracture)  Others  Arthroscopic Procedure  Rotator cuff  ACL reconstruction  Other  Other | **26 (41%)**  12 (19%)  7 (11%)  5 (8%)  1 (2%)  1 (2%)  **24 (38%)**  6 (10%)  5 (8%)  4 (6%)  4 (6%)  5 (8%)  **12 (19%)**  4 (6%)  7 (11%)  1 (2%)  **1 (2%)** |
| **Journal of publication**  *Journal*  Specialised  Generalist  Cochrane | 46 (73%)  14 (22%)  3 (5%) |
| *Top 10 impact factor*  No  Yes | 45 (71%)  18 (29%) |

RCT, randomized controlled trial; ACL, anterior cruciate ligament
